# Requirements for Creating a Position for Community Health Nursing Within the Iranian Primary Health Care System: A SWOT Analysis

**DOI:** 10.3389/fpubh.2021.793973

**Published:** 2022-01-13

**Authors:** Aazam Hosseinnejad, Maryam Rassouli, Simin Jahani, Nasrin Elahi, Shahram Molavynejad

**Affiliations:** ^1^Student Research Committee, Nursing and Midwifery School, Ahvaz Jundishapur University of Medical Sciences, Ahvaz, Iran; ^2^Cancer Research Center, Shahid Beheshti University of Medical Sciences, Tehran, Iran; ^3^Nursing Care Research Center in Chronic Diseases, Ahvaz Jundishapur University of Medical Sciences, Ahvaz, Iran

**Keywords:** community health nursing, primary health care, SWOT analysis, Iran, policy

## Abstract

**Background:** Accepting community health nursing in the primary care system of each country and focusing on creating a position for community health nurses is of significant importance. The aim of this study was to examine the stakeholders' perception of the requirements for establishing a position for community health nursing in the Iranian primary health care system.

**Methods:** This qualitative study was done using 24 semi-structured interviews conducted from May 2020 to February 2021 in Iran. The participants were selected through purposive sampling and consisted of nursing policy makers, the policy makers of the Health Deputy of Ministry of Health, the managers and the authorities of universities of medical sciences all across the country, community health nursing faculty members, and community health nurses working in health care centers. After recording and transcribing the data, data analysis was performed in MAXQDA10 software, using Elo and Kyngas's directed content analysis approach and based on WHO's community health nursing role enhancement model. The statements for each main category were summarized in SWOT classification. To examine the trustworthiness of the data, Lincoln & Guba's criteria were used.

**Results:** By analyzing the interviews 6 main categories identified consist of creating a transparent framework for community health nursing practice, enhancing community health nursing education and training for practice in the primary health care system and community settings, seeking support, strengthening the cooperation and engagement among the key stakeholders of the primary health care system, changing the policies and the structure of the health system, and focusing on the deficiencies of the health system. Each main categories including the subcategories strengths, weaknesses, opportunities and threats (SWOT).

**Conclusions:** Based on the participants' opinions, focusing on the aforementioned dimensions is one of the requirements of developing a position for community health nursing within the Iranian PHC system. It seems that correct and proper implementation of these strategies in regard with the cultural context of society can help policymakers manage challenges that prevent the performance of community health nursing in the health system.

## Introduction

The health system all around the world, including Iran, has undergone many changes during the last 20 years (1, 2) and will have encountered serious challenges by the year 2050 (3). Lifestyle changes will increase life expectancy, followed by an increase in the incidence and the prevalence of non-communicable diseases (4–8). The emergence of new diseases and pandemic crises such as COVID-19 will pose new challenges to the health systems of different countries (9, 10). On the other hand, adopting approaches to reduce the length of hospital stay, increasing outpatient surgeries, focusing on health promotion, and the desire to reduce costs will lead to a shift in paradigms, transferring health care provision from hospitals to community settings (11–13). The emerging challenges will focus on the need to change, enhance, or reform the health system (14, 15). Due to the important role of family and community in disease management and health promotion, health systems around the world will change into community-based nursing care (5, 16). Since community health nurses play an important role in supporting the transfer of hospital care to home and health centers such as comprehensive health centers, in order to address the community needs and provide cost-effective care, community health nursing services should be used (17, 18).

Community health nurses play a key role in providing quality primary health care and universal health coverage (19, 20). Nurses can take leadership roles and provide direct health care based on patient, family and health system's priorities in primary care. Care seekers are also satisfied with the services provided by community health nurses (21). Community health nurses take roles in the 4 areas of primary health care including *preventive care* (health promotion, education), *management of chronic diseases* (coordination of care, control and assessment, rehabilitation), *practical actions* (the health of children and the elderly, midwifery care), *involvement in health policymaking* (developing policies, planning, evaluation and supporting national programs in accordance with the situations, requirements and priorities) (22).

Community health nursing has grown significantly in the health system of all developed countries around the world (23). According to the WHO, more than 42% of community health nurses in the United States work in health centers. Given the fact that many actions in primary health care do not require a physician's knowledge or skills, the tendency to use community health nurses for developing the capacity of primary health care is on the rise. Replacing a physician with a nurse is a strategy that will improve the accessibility, effectiveness, and quality of health care (17, 24). In some European countries, physician-based and hospital-based approaches are replaced with community health nurses and these nurses provide health care and services to the members of the community (25, 26). Among European countries, Finland has the lowest rate of physician-patient contact, and a large number of community health nurses provide services in the health system instead of physicians. In Ireland, based on an efficient primary health care model, more integrated services at all levels of prevention, especially the first level, are provided by community health nurses to people of all ages from infants to the elderly. There are also community-level clinics directed by community nurses for providing care and prescribing medication (27). In the United Kingdom, a model exists for involving community health nurses in primary health care, in the form of clinic management, the provision of health care, education and counseling services, and even the competence to prescribe medication (15).

In Iran, the courses of community health nursing and epidemiology were included in the undergraduate curriculum after community-based and community-oriented nursing disciplines were developed in 1985 by policymakers. However, community health nurses currently encounter obstacles and challenges in offering specialized services in the form of specialty job descriptions developed by the Ministry of Health (28, 29). Currently, comprehensive urban health centers are run by graduates and associates in family health, environmental health, occupational health, disease control, and midwives and local health centers, by Behvarzes (rural health care workers), in order to provide health services. In these centers, health services are provided sporadically (28) and no effective strategies in line with community needs are implemented for care provision (30, 31). On the other hand, neither following up discharged patients and vulnerable groups such as chronic patients, the elderly, pregnant women, and infants at the community level, nor offering home visits with the aim of promoting health is done at comprehensive health centers. Despite the fact that home care services are one of the most important components of the health system, they are not yet institutionalized within the structure of the primary health care system (5). Furthermore, for addressing the challenges of the health system stated in the 6th Development Plan, the Iranian Parliament urged the Ministry of Health to provide a *comprehensive health services system* by prioritizing health and prevention over treatment and to offer primary health care by focusing on the referral system, family physician program, and the provision of home-based and community-based nursing care. The most important action taken by the Nursing Deputy of the Ministry of Health for addressing challenges against the establishment and development of home-care and nursing consultation centers was the formulation of regulations for hospices and long-term care centers (4).

Community health nurses use comprehensive approaches, as well as clinical and managerial knowledge and skills, to lead and manage care and coordinate the continuum of care transfer from hospital to home and the community. They also have the competence to coordinate the care team and interdisciplinary participation, manage patient transfers from hospitals and medical centers to comprehensive health centers, and perform their follow-up at the community level. Therefore, these services can be assigned to them (32). In terms of cost-effectiveness, various studies show that providing community-based care by community health nurses instead of hospital care leads to improved health and quality of care, chronic disease management, patients' access to community-based social services, and patient satisfaction, which in turn results in reduced ER visits and hospitalization, eventually decreasing treatment costs. In several US states, including Michigan, the community-based care delivery model has been implemented resulting in reduced overall health costs (18, 33).

Considering the effectiveness of analyzing the stakeholders' perceptions in policy-making, management and development of the health system (34), it is important to examine their views to investigate the weaknesses, strengths, opportunities and threats of community health nursing in order to identify the necessary measures for reforming the primary health care system, which is why SWOT model was used in the present study. Models can help researchers with identifying, describing, explaining or predicting (35). SWOT is a simple conceptual framework that can be used by individuals, groups, teams, and organizations providing health care (36). The aim of this study was to explain the stakeholders' perception of the requirements for establishing a community health nursing position within the Iranian primary health care system.

## Methods

### Study Design

This is a qualitative study using directed content analysis.

### Study Settings and Participants

Twenty-four subjects participated in this study. The researcher first interviewed the key informants, namely nursing policymakers, the policymakers affiliated to the Health Deputy of the Ministry of Health, the health managers and the authorities of the Iranian universities of medical sciences. Then interviews were conducted with other participants, including community health nursing faculty members and community health nurses working in health centers and various provincial deputies. The participants were selected through purposive sampling. The inclusion criteria for the participants consisted of having experience in policymaking and decision-making in the field of health and community health nursing, and voluntarily participation in the research. The time and the place of the interviews were fixed according to the participants, at their own workplaces and offices. It should be noted that due to COVID-19 pandemic conditions, some interviews were conducted via Skype or Whatsapp.

### Data Collection

The research data was collected using in-depth semi-structured interviews conducted from May 2020 to February 2021. Each interview took 30–70 min. The interviews continued until data saturation was achieved, or in other words, until new data did not modify or further develop the model and no new classes were created (37). Prior to the interviews, the interview questions guide was developed based on the policies of WHO model (20) separately for each participant group in order to ensure the comprehensiveness of the collected data. The questions included “According to your experiences, what is the position of community health nurse in Iranian health system?,” “How can we define a position for community health nurses according to their duties regarding public health promotion?,” “What are the opportunities in and barriers against providing community-based nursing services in Iran?,” “What are the executive strategies for nurses to enter the network system?.” Furthermore, in order to collect more information during the interviews and clarify the content, exploratory questions were asked such as “Can you explain more? Can you give an example?”

### Research Framework

The present study was designed and implemented based on the model “Enhancing the Role of Community Health Nursing for Universal Health Coverage” (WHO 2017). This model is a guide, a framework, and a strategy to strengthen and enhance the role of community health nursing with the aim of universal health coverage, which is considered a comprehensive action plan focusing on national and local strategies. According to the evidence obtained from a WHO study during 2010–2014, community health nursing is regarded as an important component in primary health care in 22 countries affected by a shortage of service providing manpower. In this model, four major policies were proposed to manage the challenges of community health nursing and promoting the role of community health nurses (20). The policies include *the development of a clear framework for community health nursing practice, promoting the education and training of community health nurses for working in the primary health care system and community settings, strengthening cooperation and partnership among the major stakeholders of primary health care system*, and *the development of comprehensive support plans for community health nurses in various countries*.

### Data Analysis

The data was collected and analyzed simultaneously. Data management was done using MAXQDA-v10 software (38). Data analysis including preparation, organization and reporting was done as proposed by Elo & Kyngas (39, 40) (Table 1). An example of data analysis is shown in Table 2. SWOT (Strengths, Weaknesses, Opportunities, and Threats) analysis was carried out through the content analysis of interviews. The statements of each main category of the study were integrated into SWOT classes (Table 3). The strengths and weaknesses are the internal aspects of the professional position of community health nursing in the Iranian primary health care, related to nursing schools and the nursing system, while the opportunities and threats include external environmental factors other than colleges and nursing system. An example of SWOT analysis results is displayed in Table 4.

**Table 1:** The process of qualitative data analysis based on Elo & Kyngas' Method.

Data preparation	Selecting the unit of analysis	After turning the interviews into texts, the manifest content (such as interview text) and latent content (non-verbal behavior of participants) were analyzed and semantic units were identified.
	Finding a logical connection between data and the topic in general	The interview texts were reviewed several times by the researcher, and the researcher was constantly engaged with data for a long time until data saturation was achieved.
Data organization	Creating an analysis matrix	At this stage, a unconstrained matrix was created and the following were extracted as main categories: (1) developing a transparent framework for community health nursing practice, (2) enhancing community health nursing education and training for practice in the primary health care system and community settings, (3) strengthening cooperation and engagement among the major stakeholders of the primary health care system, and (4) the development of comprehensive support programs for community health nurses.
	Data extraction from content based on classes	The possibility of placing generic categories in the main categories of the matrix or the formation of new categories was investigated, based on conceptual and logical connections.
	Categorization	The number of codes decreased by the merging of similar codes into more general codes, consequently forming the generic categories.
	Classification	The created categories were classified based on the similarities and differences and similar categories were merged.
	Abstraction	The categories discovered in the initial main categories were placed into the analysis matrix. In case of inconsistency with the existing classes, new main categories were created by merging similar classes (new classes in the current study: *changing the policies and the structure of the health system* and *focusing on the deficiencies of the health system*)
Reporting		The sampling process, the participants' characteristics, data collection, data analysis, and the analysis of each class are thoroughly reported under *Findings*.

**Table 2:** An example of data analysis.

**Main category**	**Generic categories**	**Subcategories**	**Primary codes**	**Quotation**
promoting the education and training of community health nurses for working in the primary health care system and community settings	Modifying the undergraduate education system	The necessity of nursing education system's being community-based	- The individual-based undergraduate education system - Disease-based approach in the education - Not being prepared for community-based services	“Practically, the undergraduate education system is not community-based, but is basically individual-centered, and even patient-centered, which still focuses on diseases. Not much attention is paid to the subject *individual care seeker* and the main focus is on the disease-based approach. When you teach someone in accordance with such a system, in my opinion, one is not prepared to offer community-based services.”

**Table 3:** A summary of the statements of the SWOT analysis based on 4 subcategories.

**Main category**		
Creating a transparent framework for community health nursing practice	**Strengths**	**Weaknesses**
	Community health nurse's ability to manage and follow up on chronic patients with regard to the previous experiences of the holistic view of community health nursing	Not recruiting community health nurses for supplying community health services
	**Opportunities**	**Threats**
	Redefining the duties of comprehensive health centers in the development plan, and assigning a health care title for nurses	Considering a therapeutic role for nurses working in health sectors and health managers' unfamiliarity with nurses' capabilities in the field of health The lack of need for developing a position for community health nurses in the primary health care system from the perspective of the senior managers of the Ministry of Health
Enhancing community health nursing education and training for practice in the primary health care system and community settings	**Strengths**	**Weaknesses**
	Thorough and comprehensive theoretical framework for the community health nursing graduate program	Deficiencies in offering practical courses in undergraduate and post-graduate community health education programs Deficiency in practical courses due to inappropriate fields Low quantity and quality of community health nursing instructors Poor community-based and community-oriented nursing education Community health nursing curriculum's not being tailored to community health needs
	**Opportunities**	**Threats**
	Offering a previously-developed design to present post-graduate courses for nurses' employment in health centers Offering specialty training to community health nurses in undergraduate programs	Considering a therapeutic role for clinical disciplines such as nursing by the Ministry of Health
Seeking Support	**Strengths**	**Weaknesses**
	The existence of nursing boards and associations effective in seeking support	Community health nursing leaders' not taking actions to introduce this discipline and not publicizing information in this regard Lack of interactions with the policymakers of the Ministry with the aim of presenting community health nurses' capabilities Insufficient measures taken by the nursing system organization in support of community-based nursing
	**Opportunities**	**Threats**
	The existence of a position and deputy for nursing (Nursing Deputy)	Public unawareness of community health nursing and the capabilities of community health nurses Highlighting the therapeutic role of nurses in the media
Strengthening the cooperation and engagement among key stakeholders of the primary health care system and	**Strengths**	**Weaknesses**
	**-**	Separate actions taken by various departments in universities of medical sciences The lack of common courses between Health and Nursing departments in post-graduate education
	**Opportunities**	**Threats**
	Nursing Deputy's power and capacity in establishing communication with other deputies of the Ministry, especially the Health Deputy	The lack of interdisciplinary interaction between health disciplines Tribalism in the health system
Changing the policies and structure of the health system	**Strengths**	**Weaknesses**
	Community health nurses' interest in working in health sector	Not being motivated to work in the Family Physician Plan and health sector due to insufficient salaries and payments The lack of an effective nursing leadership across the country Nursing managers' taking no actions in order to extend the boundaries of nursing practice The shortage of nursing clinical workforce Barriers against employing nurses in the health sector Low enrollment capacity in community health post-graduate programs
	**Opportunities**	**Threats**
	The existence of job titles for nurses and health care giver in the PHC Operational objectives of Nursing Deputy regarding holistic nursing The focus of senior managers of the health system on legalization of nursing services at the community in the form of home-care and consultation centers	Not giving health priority over treatment in the health system and focusing on the secondary prevention Conflicts of interest with other medical disciplines The lack of power to change and modify the network system Insufficient funding and budgets for employing community health nurses
Focusing on the deficiencies of the health system	**Strengths**	**Weaknesses**
	Better nursing responsiveness to the public's health needs Empowering nurses for the coordination of medical and care plans	High workload of caregivers and low manpower-population ratio Routinized activities of comprehensive health centers and ignoring the personal needs of care seekers The lack of public trust in care and consultation offered by health caregivers regarding chronic diseases
	**Opportunities**	**Threats**
	The possibility for health system policymakers to use nursing care plan at home in order to employ community health nurses	Deficiency in the service delivery system and the referral system and not covering all areas of prevention Entrusting health policymaking management to a conscripted physician who is not familiar with people in comprehensive health centers health center physicians' focusing on therapeutic measures instead of providing health promotion services Behvarzes' not addressing people's health needs

**Table 4:** Main categories, generic categories, and sub-categories extracted from the content-driven analysis of the interviews.

**Main categories**	**Generic categories**	**Subcategories**
Enhancing the community health nursing	Creating a transparent framework for community health nursing practice	Explaining the position of community health nurse
		Community health nursing service areas
		Job descriptions for community health nurses in comprehensive health centers
	Enhancing community health nursing education and training for practice in the primary health care system and community settings	Reforming the undergraduate education system
		Reforming the community health nursing post-graduate program
		Flaws in the educational system input
	Seeking support	The necessity of establishing a communication channel with the public
		The necessity of receiving support from nursing policymakers
	strengthening the cooperation and engagement among key stakeholders of the primary health care system	The Key stakeholders' role
		The necessity of interdisciplinary cooperation in education
	Changing the policies and structure of health system	The barriers against the provision of community health nursing at the level of Health and Treatment Deputy
		The barriers against the provision of community health nursing at the level of Education Deputy
		The barriers against the provision of community health nursing at the community level
		The necessity of effective performance of community health nursing policymakers
		The necessity of strengthening community health nursing institution
	Focusing on health system deficiencies	Ignored services of the health system
		Deficiencies in the network system's functioning
		Deficiencies in the healthcare team's performance

### Rigor and Trustworthiness

In order to enhance the rigor and the trustworthiness of the data, the four indicators credibility, dependability, confirmability, and transferability of Lincoln and Guba were used (41). Data credibility was determined through long interaction with data, member check, peer review, and reviewing the interview texts by the participants. Data dependability was ensured through responsiveness. In other words, the process of research and data analysis was examined by an external supervisor familiar with qualitative research. In order to ensure confirmability, all the steps of research are presented and explained in detail. Data transferability was achieved by providing a deep and rich explanation of the findings and the maximum amount of sample variance.

### Ethical Considerations

This study was approved by the Ethics Committee of Ahvaz Jundishapur University of Medical Sciences (IR.AJUMS.REC.1398.874). Ethical considerations in this study included the voluntary nature of research participation, the explanation of research objectives to the participants, and ensuring their anonymity, their right to withdraw from the study, and the confidentiality of the data.

## Results

Twenty-four subjects participated in this research, including 6 nursing policy makers and policy makers of the Health Deputy of Ministry of Health, 7 health managers and authorities of universities of medical sciences, 8 community health nursing faculty members, and 3 community health nursing working in health centers. The mean age and working experience of the participants were 53.5 ± 9.83 and 24.91 ± 6.09 years, respectively. 54.2% of the participants were male, and 45.8%, female. The inclusion criteria for community health nursing faculty members consisted of having at least 10 years of working experience in educational environments. The inclusion criteria for policy makers consisted of managerial experience in the health system in the field of health or nursing. Community health nurses had to have at least 5 years of working experience in health centers.

After continuous analysis and comparison, 780 codes were extracted, and 76 subcategories, 18 generic categories, and 6 main categories were identified. The main categories included *creating a transparent framework for community health nursing practice, enhancing community health nursing education and training for practice in the primary health care system and community settings, seeking support, strengthening the cooperation and engagement among the key stakeholders of the primary health care system, changing the policies and the structure of the health system*, and *focusing on the deficiencies of the health system*, each of which consisting of 4 subcategories: *strengths, weaknesses, opportunities*, and *threats*.

### Creating a Transparent Framework for Community Health Nursing Practice

In order to create a transparent framework for community health nursing practice, it is necessary to make huge policies in the Ministry of Health. A transparent framework for community health nursing practice includes the explanation of a position for the community health nurse, a scope for their services, and a clear job description for them.

#### Strengths

The participants referred to the community health nurses' ability in managing and following up on patients with chronic diseases at the community as a strength of the health system. “In my opinion, today, we offer individual-level health care through Behvarzes and caregivers, but in order to adopt a community-oriented to the management of chronic diseases, we need a number of capable people at a higher level, such as these community health nurses who can be employed in comprehensive health centers,” the director of the department of non-communicable diseases in one of the provinces said in this regard, “In old diabetes clinics, the presence of a nurse, and using teamwork was a successful experience in the management of diabetic patients.”

#### Weakness

The participants mentioned the recruitment of community health nurses only in academic and clinical areas, the inability of nursing managers to employ community health nurses in the network system, and community health nursing graduates' not taking actions to develop a position for their own discipline. “Officially, no position exists for community health nurses in the charts of comprehensive health centers, in other words, they have no place. The only position for community health nursing is acting as a faculty member in the health departments of nursing schools.”

#### Opportunities

Considering a the nurses at the level of PHC in the health promotion plan was considered, by the participants, as an opportunity to create a framework for community health nurse practice. “All across the country, in all urban and rural centers we have nursing titles for health nursing experts in the health organizational chart,” the executive director of the Health Deputy in one of the provinces said, “if you take a look at the health chart, you will realize that the nurse is regarded at the level of PHC, and nursing managers can define this position for community health nursing through consulting with the Deputy Minister of Health.”

#### Threats

Not needing to create a position for community health nurses in the primary health care system from the perspective of the managers of the Ministry of Health is one of the most important threats to creating a framework for community health nursing practice. “Community health nursing is a concept, up to 80% of which is currently realized by Behvarzes and healthcare providers. Given the current state of the health system, there is no need to create a position for community health nurses. Such workforce should not be placed at higher levels,” the Deputy Minister of Health said in this regard.

### Enhancing Community Health Nursing Education and Training for Practice in the Primary Health Care System and Community Settings

Enhancing the education and training of community health nurses in accordance with the needs of the society is one of the goals of the educational system. To this end, it is necessary to reform the educational system in accordance with the needs of society and change the educational approach from being hospital-based to being community-based. It is also necessary to focus on the inputs of the educational system, which includes a sufficient number of experienced community health nursing faculty members and appropriate fields of education in community health nursing.

#### Strengths

The participants referred to thorough and comprehensive theoretical frameworks in community health nursing post-graduate program. “There are no theoretical issues in community health nursing post-graduate curriculum and it is even being reviewed, because it should be reviewed every five years. But the implementation of the program is very important, and it is the teacher who plays the key role in this regard,” a member of the community health nursing faculty said on the matter.

#### Weaknesses

The participants referred to the inefficiency of practical courses in community health nursing curriculum due to improper fields and the weakness of community-oriented education in the nursing education system as the most important weaknesses. “The environment is not suitable for the practice of health traineeship. Trainees mostly end up in health centers because the interdepartmental systems are not ready for the community health nurses home visits, which makes the trainings impractical. The only thing that works is teaching in the classroom. At best, our nurses are well-trained for clinical purposes,” one of the community health nursing faculty said regarding the importance of teaching practical community health nursing courses.

#### Opportunities

The prticipants regarded the possibility of training community health nursing at undergraduate level as an opportunity. “It would be excellent if nursing schools accept to train some nurses, from the beginning at the undergraduate program, tailored to the characteristics of community health nursing including the provision of environmental health services, health education, nutritional health, maternal health, fertility and immunization,” said the Deputy Minister of Health in this regard, “In other words, it will be good if there is a bachelor's degree program in community health nursing. If we modify the education system in this way, our nurses will no longer have many options to be able to choose from among angiography, pediatrics, surgery and health at the same time and decide on their own. In that case, we have the workforce and we can organize it.”

#### Threats

The participants referred to the need to reform the Ministry of Health's educational perspective of clinical disciplines and argued that education should be revised with the aim of becoming community-oriented. “In general, the therapeutic perspective is dominant in the Ministry of Health, which means that the main problem lies in the education system. Treatment perspective is dominant, not health and community-oriented views. Nurses spend little time in health departments, while they spend a lot of time in hospitals as trainees, and they even interpret ECG better than a physician. But the same nurses have no knowledge when it comes to working at the community level, and cannot provide simple trainings to people,” said the Deputy Minister of Health of one of the provinces on the matter.

### Seeking Support

Seeking support requires the creation of a communication channel with the public in order to increase public awareness of community health nursing discipline and see support from nursing policymakers to introduce this profession to the public.

#### Strengths

The participants considered the existence of nursing boards and associations to be effective in supporting community health nursing.

“Seeking support requires actions taken by nursing institutions such as nursing boards and scientific nursing associations to state the capabilities of community health nurses and allow them to demonstrate their capabilities to the health system and the public. More efforts should be made in this regard and the Scientific Association of Community Health Nursing should be more active,” said a member of the community health nursing faculty member on the topic.

#### Weaknesses

The participants referred to the need to establish a communication channel between community health nurses and the public for support, and believed that people do not know community health nursing at all. “The leaders of community health nursing do not try to introduce the discipline either at the university level or to the people of the community. However, if they define themselves as the community health care institution and the provider of public health services, people will seek health services from them. Introducing the discipline can be very effective in seeking support because once the discipline is recognized, organizations will make a recruitment call. Introduction should be done hierarchically downwards at different levels,” said one of the nursing board members in this regard, “Many in the Ministry of Health are, themselves, unaware of the existence of this type of workforce with such capabilities, because everyone we trained was recruited by the clinical and academic nursing community.”

#### Opportunities

The participants introduced the position of Nursing Deputy as one of the most effective opportunities for strengthening the community health nursing institution, and believed that it plays a significant role in the entry of nurses into the network system. “The Nursing Deputy itself is a good opportunity, where recently, PhD graduates have been working. We also have individuals whose experience can be helpful,” said a community health nursing faculty member in this regard.

#### Threats

From the participants' perspective, the most important threat is highlighting the therapeutic role nurses in the media. “Highlighting the therapeutic aspect of nursing by the Islamic Republic of Iran Broadcasting on occasions such as the Nurses Day, has led to the public's unawareness of community health nursing and the capabilities of this discipline in providing health and preventive services,” another community health nursing faculty member stated in this regard.

### Strengthening the Cooperation and Engagement Among Key Stakeholders of the Primary Health Care System

Strengthening the cooperation and engagement among key stakeholders requires actions to be taken by Nursing and Health deputies, designing programs to encourage public engagement, and the interaction of community health nursing professors with the professors of health departments.

#### Weaknesses

The participants regarded the departments' lack of knowledge of each other at a medical university, and the lack of interdisciplinary engagement among different disciplines of the Ministry of Health as the most important weaknesses. “The most important issue in our country is that actions are taken dispersedly. In other words, academicians in one department at a university are not aware of another department at the same university. They may not know about each other and what others are capable of. When we get acquainted with a colleague in another department, we will get to know each other's capabilities and what our fields of study have in common,” a nursing board member said in this regard.

#### Opportunities

The participants mentioned the Nursing Deputy's capacity and capability in communication with other deputy ministries, especially the Health Deputy, with the aim of strengthening interdisciplinary cooperation. “Today, with the help of competent individuals, we expect the Nursing Deputy to take an effective step in providing health services through interaction and communication with the Health Deputy with the aim of strengthening the cooperation between community health nurses and the health staff,” one of the nursing policymakers said in this regard.

#### Threats

The participants considered interdisciplinary interaction necessary for strengthening cooperation and engagement. “Unfortunately, in health-related disciplines, no interdisciplinary interactions exist and our country's health system is governed through trade unionism and tribalism. As long as there is unionism, we should not expect inter-professional cooperation and engagement,” another nursing policymakers stated on the matter.

### Changing Policies and the Structure of the Health System

It is necessary to change the policies and the structure of the health system in order to prioritize health over treatment, change the structure of the network for community health nurses' entry to the primary health care system, resolve infrastructure issues (for instance, through the approval of supportive laws for community health nurses), issue permits for the establishment of community health clinics according to the structure of the referral system, and facilitate the insurance coverage of community health nursing services.

#### Weaknesses

The participants considered the community health nurses' reduced motivation to work in the health departments compared to the treatment sector as a weakness, and referred to the need to reform the payment system in the health sector. “Many senior nurses are reluctant to work with us due to low salaries and financial benefits in the health sector. Currently, a nurse in the treatment sector has a monthly income of about 10 million Tomans, which is reduced to 3 or 4 million Tomans when it comes to health, a very low amount,” the executive director of the health deputy of one of the provinces said in this regard, “The job that a nurse gets in a hospital is very, very different from the job that we do in the health sector. Thus, nurses prefer to work with their bachelor's degree rather than providing health services with a master's degree. Another problem is the shortage of manpower. For this reason, the Treatment Deputy recruits all nurses, even community health nursing postgraduates, and does not give them to the Health Deputy.”

#### Strengths

The participants stated community health nurses' interest in working in the health sector as one of the strengths, and believed by providing proper infrastructure this advantage can be used to benefit from the capabilities of community health nurses.

“Many nurses entering the community health discipline liked working in the health sector because of greater independence compared to clinical jobs and were interested in working with people at the community level,” one of the community health nurses working in a health center said on the subject.

#### Threats

From the participants' perspective, in the health care system, hospital care takes precedence over community-based services. “It is true that we call it the Ministry of Health, but we do not see the priority of health over treatment. Our system is not health-oriented, but treatment-oriented and patient-oriented, because there is a conflict of interest,” another nursing policymaker said, “For instance, in Shahrak-e Gharb, there are lots of private hospitals and more are still being launched, but not even one preventive clinic at the community level can be found. This shows that we do not access preventive procedures and cannot take preventive measures, one of the requirements of which is community health nursing.”

#### Opportunities

The goal of making nursing community-oriented was considered as an opportunity by the participants. “Fortunately, the Deputy's strategy is community-based. On the other hand, the governmental authorities and some of the ministers have legalized the provision of nursing services for patients at the community in the form of home care and consultation centers. But we believe that this position should also exist in preventive areas in the form of nursing services in clinics, health centers, and health complexes.” one of the nursing policymakers said, in this regard.

### Focusing on the Deficiencies of the Health System

This class considers the flaws in the functioning of the network system and the performance of the health team as the deficiencies of the health system.

#### Weaknesses

The participants regarded the disproportionate manpower-population ratio, and the routinized work of the staff of comprehensive health centers without considering the individual needs of the care seeker as significant weaknesses.

“Unfortunately, due to their high workload, our community health workers are not able to provide care and follow up on patients with chronic diseases like diabetes and hypertension in proper way. People do not trust health centers with the care provided for chronic diseases, because measuring blood pressure and weighing patients is also done by our health care providers. Since people receive better services from private centers than the ones we offer, they won't come to us in health centers.” said the executive director of the Health Deputy in one of the provinces.

#### Strengths

The better responsiveness of community health nurses to the public's health needs, according to nurses' education in regard with diseases and care compared to the midwifery and health students was seen as a strength by the participants.

“In our health centers, a community nurse according to their knowledge, can very well-fulfill their duties in health promotion, education and prevention based on the needs of the people and follow-ups of chronic patients at the community, as well as follow-up of the patients discharged from the hospital.” one of the health policymakers of the Ministry of Health said in this regard, “even one of our BSc graduate nurses has the ability to know the community and has the power to establish a communication with the workforces lower in hierarchy and those higher (i.e., physicians), which is a great advantage for the nurse, making them capable of making the necessary coordination in the field of health. We can even have a community nurse provide home care, and accordingly, we have to define *community nurse* based on the population covered by that health center.”

#### Opportunities

Participants believed that health system policymakers should use the home care nursing plan to employ community health nurses in this field and develop the profession in various areas of the community.

“Home nursing care and consultation plan was a very good plan that health system policymakers, both in nursing and health, could use to provide services to people at the community level by employing specialist nursing workforce, especially community health nurses and even nursing experts. But unfortunately, this plan has not been implemented well so far. The work was done to some extent, but it wasn't further developed. The Nursing Deputy can use these centers as an opportunity.” one of the nursing policymakers said in this regard.

#### Threats

Defects in the country's service delivery system and its not being fully based on the PHC structure as well as the flawed classification and referral system were regard, by participants, as the most important threats.

“The service system in our country is incompletely based on the PHC structure. PHC means the classification of services and the referral system. On the other hand, in case service levels exist and control each other well, we will actually be able to provide appropriate and timely services to the public. Now our nurses have become more specialized, but their only duty is to respond to patients at the time of hospitalization, and they do nothing at the community level before or after hospitalization.” one of the health policy makers of the Ministry of Health said on the matter.

## Discussion

The present study explained the professional position of community health nursing in the Iranian PHC system from stakeholders' perspective. The extracted areas for the position of community health nursing in the Iranian health system from stakeholders' perspective were classified in 6 main categories including *creating a transparent framework for community health nursing practice, enhancing community health nursing education and training for practice in the primary health care system and community settings, seeking support, strengthening the cooperation and engagement among key stakeholders of the primary health care system, changing the policies and structure of the health system* and *focusing on the deficiencies of the health system*. It is worth noting that 4 main categories of this study are consistent with WHO's Community Health Nursing Role Enhancement Model and the main categories *changing the policies and structure of the health system* and *focusing on the deficiencies of the health system* were obtained from the findings of the current study.

The first main category was *creating a transparent framework for community health nursing practice*. In order to create a transparent framework for the practice of community health nursing, a role should be defined for them in the health system and a position, in PHC (20). Therefore, one of the most important infrastructural issues is the development of a position and job description in the organizational chart for community health nurses in comprehensive health centers. Currently, in Iran, the services offered by community health nurses are mainly provided at the third level and in hospitals, because no position exists for community health nurses in comprehensive health centers (4, 28), while in developed countries, the first level of people's contact with the health system is through community health nurses (27). Numerous studies have addressed the need to explain the position of community health nurses in the country's health system and its importance in promoting health and reducing costs (5, 13, 42). Therefore, creating job opportunities for community nurses is among the infrastructure necessary for the provision of nursing services at the community (28).

The second main category was *enhancing community health nursing education and training for practice in the primary health care system and community settings*. One of the important areas of the WHO's Community Health Nursing Role Enhancement Model is the implementation of training programs to empower community health nurses, interdisciplinary training and their continuous professional promotion with the aim of improving the quality of health services provided in health centers (20). Participants referred to the poor community-based education in nursing and argued that that undergraduate curriculum needs to be revised to become community-based. In recent years, due to the increased burden of chronic diseases at the community, nursing education experts around the world aim to make changes in the traditional hospital-based curriculum and approaches, in order to redesign the curriculum with a focus on community-based care (14). The study by Cheraghi et al. also referred to Iranian nursing graduates' insufficient skills and their negative attitudes toward quality care at the community level as a result of being trained through hospital-based services (43). The educational system of medical universities is not compatible with PHC and curriculums are not tailored to needs, and consequently, university graduates do not possess the necessary skills to face the problems. Therefore, academic education courses should be enhanced and the trainings should be provided in line with the PHC (30, 31). Participants also mentioned the specialization of undergraduate nursing program with a focus on training health nurses tailored to the needs of the health system. In their study, Jarrín et al. stated that the beginning of a community health nursing education program, in the first months of students' arrival, in the form of lectures, introductory textbooks and simulation regarding home-care and community-based care will significantly impact their beliefs and attitude toward community-based nursing, because the traditional curriculum has undermined the value of community activities and home care in students' mind (14).

The third main category covered the development of comprehensive support programs for community health nurses in Iran. In order for support programs to influence the policymakers, a proper understanding of the issues affecting community health nurses and how they relate to people's health at the community is essential. It is necessary to increase public understanding of community health nursing and support for it through the use of mass media (20). Participants referred to the inaction to introduce the community health nursing discipline and its potentials at the academic and community level as an important weakness and considered the development of a communication channel for community health nurses necessary in order to enhance public awareness through the media (30). The study by Heydari et al. also emphasized on preparing the society and increasing the level of public awareness for receiving community-oriented nursing services (28). Poor public perception of their own rights in the health sector is one of the barriers mentioned by the participants. Other studies have also focused on empowering the society to demand health from the government because the members of society do not have the feeling of being a part of the health system and are reluctant to participate in health programs (30). In other words, the lack of discourse between nursing managers and policymakers of the Ministry of Health with the aim of publicizing the capabilities of community health nurses is another weakness been mentioned in this study. The study of Yazdani et al. also showed that there is no mutual discourse between nursing institutions and other institutions of the health system and as a result, the institutions of the health system are not aware of nursing and its master's degrees specialties (44). From participants' perspective, the Nursing Deputy is one of the most effective opportunities to seek support for community health nurses to enter the network system. In response to challenges against the health system, the Ministry of Health established the Nursing Deputy in 2013 (4).

The fourth main category extracted from the study was *strengthening cooperation and engagement among key stakeholders of the primary health care system*. Given the importance of interdisciplinary cooperation, WHO emphasizes the need for the health system to be assessed in each country by its authorities and policymakers in terms of the challenges against interdisciplinary cooperation and engagement (20). From participants' perspective, one of the most important barriers against cooperation and engagement is the sporadic performance of various departments of medical universities. Other studies also refer to problems in interdisciplinary cooperation among different health institutions in the country, ignoring the principle of public engagement and the lack of a clear mechanism for it and failure to utilize the potentials of NGOs and charities that indirectly affect health (29, 30). Intersectoral collaborations in PHC are achieved haphazardly in some areas but they lack a predefined structure and institution (45). However, according to participants, the presence of Nursing Deputy in the Ministry of Health was a good opportunity to strengthen interdisciplinary cooperation with other departments, especially the Health Deputy, for the entry of community health nurses into the network system. In this regard, the study by Yazdani referred to the need for mutual discourse among nursing institutions and other institutions of the health system with the aim of strengthening interdisciplinary cooperation and engagement (44).

The fifth main category of the study was *changing policies and the structure of the health system*. Health care policies in each country are subject to its dominant ideology. In the eyes of health system policymakers, the nursing profession is not taken seriously and is not treated as a valuable profession. The chaos and disorder of the health system has caused people not to fulfill their real roles, and thus the position of nursing and its specialties in the health care system is unclear (44). Participants referred to not giving health priority over treatment in the health system and prioritizing secondary prevention in Iran as one of the most important threats. In line with the results of the present study, other studies have pointed to the treatment-oriented attitude of the managers of the Ministry of Health and the weakened position of health in the health system (30, 44). Participants identified conflicts of interest as another threat to the health system. Other studies suggest that treatment is more attractive than PHC, so physicians and even family physicians adopt a therapeutic approach and are reluctant to offer preventive and care programs (30, 46). From the participants' point of view, another threat is insufficient funding and budgets for recruiting community health nurses in the network system. One of the deficiencies of PHC in developing countries is greater willingness to spend on and invest in specialized and treatment levels (47). In Iran, the PHC system is funded fully by the government and the main weakness of the system is inadequate financing and inconsistency between resources and the necessary service packages. On the other hand, the salary of PHC staff is unfair, not proportionate to the way the services are provided, thus it does not encourage improved performance, quality and efficiency (30, 48).

The last main category extracted in this study was the *deficiencies of health system*. Health care based on the care seekers' needs will mainly lead to health promotion and increased client satisfaction. Thus, health care providers should pay attention to this important matter (49). In interviews with the visitors of comprehensive health centers, they pointed to the routinized care that was provided, and specifically, the middle-aged and elderly visitors were dissatisfied with the consultation and education received in regard with their underlying diseases. In line with the results of the present study, Heydari et al. also referred the provision of dispersed health services and health experts' not using effective strategies in accordance with the needs of the community (28). Services are provided at a basic level in health centers and people receive supplementary services outside the PHC system, which due to the nature of new needs, they will be difficult to address with this level of services (46).

Another finding mentioned by the participants was the flaw in the PHC-based service delivery system, i.e., failure to follow-up on care seekers at the community level before and after hospitalization as well as patients with chronic diseases or disability under home-care due to the high workload of health care providers and their lack of expertise. In Iran, PHC organizational structure does not have the necessary flexibility for making modifications proportionate to changes and encounters challenges in meeting the new needs of the population, which gradually weakens the system. Therefore, this system needs structural modifications (30, 31, 48). Another threat mentioned by the participants was Behvarzes' lack of response to public health questions due to the increased public knowledge. PHC manpower has not grown in line with the new needs and services and too many duties have been imposed on them. On the other hand, the changes in people's lives and level of knowledge have led to reduced acceptance of Behvarzes and as a result, their relationship with the people has diminished. Thus, recruiting Behvarzes with low level of education is not a good strategy in the current situation. Despite there are university graduates in most of the regions, no mechanism exists to use them (30, 48).

The present study examined stakeholders' perception of the requirements for establishing a community health nursing position in the Iranian primary health care system. Although, community health nursing is not a new concept in Iran and for more than four decades, nursing students have been trained in the field of community health and the Department of Public Health in the past was one of the four nursing departments at level of the Ministry of Health, the transformation and changes in the health system and the physicians' professional dominance have led to the expansion of hospitals. However, the analysis of strengths, weaknesses, opportunities and threats of every main category revealed obvious differences between health system management in Iran and other countries in the promotion of community health nursing. Thus, the Iranian health system have to make management plans and both large- and small-scale policies in accordance with the community health nursing. In this qualitative study, in order to improve generalizability, it was tried to achieve maximum diversity in the selection of participants in the fields of health and nursing policy-making, community health nursing faculty members and those working in health centers from different medical universities across the country as well as visitors. It is suggested that future studies investigate nursing students' perception of the professional position of the community health nursing.

## Study Limitations

Covid-19 epidemic made the situation difficult for in-person interviews due to the long distance of some professors, specialists and experts in the field of health across the country. The problem was addressed to a great extent through participants' cooperation and conducting interviews via Skype and What Sapp.

## Conclusion

Based on the participants' opinions, developing a position for community health nursing in the Iranian health system in line with the provision of community-based health care can be considered as one of the priorities of health system development. It seems necessary to establish community health nursing in the PHC system in order to address the health care needs of the society. Raising public awareness using social networks in regard with the services providable by health nurses can contribute to this goal.

## Data Availability Statement

The raw data supporting the results of this article will be available by corresponding author upon request.

## Ethics Statement

This study was approved by the Ethics Committee of Ahvaz Jundishapur University of Medical Sciences (IR.AJUMS.REC.1398.874). The patients /participants provided their written informed consent to participate in this study. Participants were also assured that the data would remain confidential and anonymous. They were also informed that they could be excluded from the study at any time.

## Author Contributions

AH, SJ, and MR: research conception and design. AH: interview with participants, writing, and drafting of the manuscript. AH, MR, SJ, NE, and SM: analysis and interpretation of data. MR, SJ, NE, and SM: review and editing. All authors contributed to the article and approved the submitted version.

## Funding

This article is a part of nursing PhD dissertation on nursing from Ahvaz Jundishapur University of Medical Sciences (NO 1398.874) which was financially supported by the Nursing Care Research Center in Chronic Diseases of Ahvaz Jundishapur University of Medical Sciences (NCRCCD-9837).

## Conflict of Interest

The authors declare that the research was conducted in the absence of any commercial or financial relationships that could be construed as a potential conflict of interest.

## Publisher's Note

All claims expressed in this article are solely those of the authors and do not necessarily represent those of their affiliated organizations, or those of the publisher, the editors and the reviewers. Any product that may be evaluated in this article, or claim that may be made by its manufacturer, is not guaranteed or endorsed by the publisher.
